# "The Hospital" to Its Readers

**Published:** 1896-10-03

**Authors:** 


### "The Hospital" to Its Readers.

The recurrence of October brings with it that reawakening, that rejuvenescence to which hospital affairs are annually subjected in consequence of their intimate alliance with the schools. Every year in October physicians, surgeons, and professors of allied and contributory sciences, come together from the four corners of the globe, after a season of holiday and mental recuperation, to undertake again the serious duties of their life work—the healing of the sick and the teaching of their art. Every year also at this time new men come to the schools; and, lastly, every year in October the great medical societies recommence their meetings for the interchange of experiences and the advancement of medical science. It is fitting then that The Hospital, always working in sympathy with its readers, should share in this annual renewal of youth. Hence it has always been our plan to make the beginning of The Hospital volume coincide with the commencement of the hospital year. This year, besides introducing various improvements in several other details, we have endeavoured to make our readers sharers in the great success which The Hospital has achieved by commencing the new volume with an entirely new fount of type. This, we hope, will help to carry out what has always been our aim, to make The Hospital a journal which shall be read with pleasure from cover to cover, and to ensure that the information which it contains shall be conveyed to its readers with the least possible strain to the eye and the least possible labour to the brain. We hope, then, that the new type in which The Hospital is now printed will facilitate that communication between mind and mind which is the very aim of literature. We also have to announce that we have removed to larger and more commodious offices. We shall in our new offices be able to offer much greater facilities to our advertisers than have hitherto been available, and we hope, by aid of the much better editorial offices which will now be at our disposal, to make the editors of The Hospital more accessible to those who are interested in medicine and in hospital administration than has hitherto been the case. In regard to the principles on which The Hospital is conducted they are simple; we would increase charity, we would remedy abuses, we would in every way enlarge the field of science and increase the dissemination of knowledge. While, however, holding with what has been proved to be useful, we would in all things recognise that the world is changing; not century by century as of old, but year by year, and even week by week, and that if medicine is to maintain and increase its position among the sciences and its hold upon the people, it must in all cases conform to the actual rather than merely appeal to tradition.

The medicine of to-day includes a due appreciation and apportionment of all those influences which make for health. Sometimes these are drugs which can appropriately be given in a bottle as of old, sometimes they involve the use of baths and waters, sometimes of massage and electricity, but directly we leave the "bottle" and the "pill-box" they involve, in a yearly growing degree, the "institution." In hospitals, in asylums, in convalescent homes, in hydropathic establishments, in homes for "cures" of various description, and in the many "private hospitals" of specialists of all degrees, people seek for health; and the medical man who refuses to inform his mind about such places and to know what is going on within them, finds himself drifting out of current modes of thought, while the doctor who still pins his faith only to his pill-boxes is lost.

Meanwhile there has grown up around the medical profession a tribe of helpers whose help can be no longer refused if medicine is to do its best for the sick. Nurses are everywhere; and it is impossible for medical men to use these nurses to the best advantage unless they are aware of the sort of knowledge they possess. All this they gain in the Nursing Supplement to The Hospital.

To the nurses themselves it is hardly necessary to say a word. They know by experience that The Hospital Nursing Mirror is the best paper for their purposes, and that if they wish to take it in for their own use they can subscribe for it at the rate of one penny a week.

For details as to the usual contents of The Hospital we would refer those who are not regular readers to the advertisement on page i., but to all our readers we would add one word about the ethical rules which we would advocate, and by which we think the conduct of medical men towards one another should be guided. They are simple, and, while according with all that is best in medical etiquette, they pass over much that is effete. They are founded on a sermon which has obtained the homage and admiration of the heathen as well as of Christendom, and may be summed up in the words, "Do unto others as ye would they should do unto you."

